# Supplementary material for: Intracranial Aneurysm Classifier Using Phenotypic Factors: An International Pooled Analysis
Source: J Pers Med. 2022 Aug 30;12(9):1410. doi: 10.3390/jpm12091410 (PMC9501769; doi:10.3390/jpm12091410)
Supplement: Supplementary file 1 [file jpm-12-01410-s001.zip › jpm-1722577-SI.pdf]

Table S1: International Stroke Genetics Consortium-Intracranial Aneurysm Groups (in alphabetical order of the cohort's name)

COHORTS	Coordinating Institution
@neurIST_EMC_NL	Erasmus Medical Center, Rotterdam, The Netherlands
@neurIST_GVA_CH	Neurosurgery Division, Department of Clinical Neurosciences, Geneva University Hospitals, Geneva, Switzerland
@neurIST_HBAR_ES	Hospital clinic Barcelona and Barcelona hospital General de Cataluna, Barcelona, Spain
@neurIST_MUP_HU	University of Pecs, Pecs, Hungary
@neurIST_UOXF_UK	Radcliffe Infirmary, Oxford, United Kingdom
@neurIST_USFD_UK	Royal Hallamshire Hospital, Sheffield, United Kingdom
ACROSS_AUS	Australasian Cooperative Research on Subarachnoid Hemorrhage Study, Australia/New Zealand
BASICMAR_ES	IMIM and Hospital del Mar, Barcelona, Spain
CCC_USA	University of Cincinnati, College of Medicine, Cincinnati, Ohio, United States of America
FIA1_USA and<br>FIA2_USA	University of Cincinnati, College of Medicine, Cincinnati, Ohio, United States of America<br>Departments of Neurology and Public Health Sciences, University of Virginia School of Medicine, Charlottesville, Virginia, United States of America
FRCAN_CA	Montreal Neurological Institute and Hospital, McGill University, Montréal, Quebec, Canada
GERFHS1_USA and GERFHS2_USA	University of Cincinnati, College of Medicine, Cincinnati, Ohio, United States of America
GOSH_UK	Stroke Research Centre, University College London Queen Square Institute of Neurology, London, United Kingdom
GSA_NL	Department of Neurology and Neurosurgery, University Medical Center, Utrecht Brain Center, Utrecht University, Utrecht, The Netherlands
HUCH_FI	Department of Neurosurgery, Helsinki University Hospital, University of Helsinki, Finland
ICAN_FR	Université de Nantes, CHU Nantes, INSERM, CNRS, l'institut du thorax, Nantes, France<br>CHU Nantes, Department of Neuroradiology, Nantes, France
JUMC_PL	Department of Neurology, Faculty of Medicine, Jagiellonian University Medical College, Krakow, Poland
KIAD_FI	Neurosurgery NeuroCenter Kuopio, University Hospital Kuopio, Finland
UCSF_USA	Department of Neurology, University of California at San Francisco, San Francisco, United States of America
UMCU2_NL	Department of Neurology and Neurosurgery, University Medical Center, Utrecht Brain Center, Utrecht University, Utrecht, The Netherlands.

Table S2: Characteristics of the different cohorts

COHORTS	Cohort basis recruitment	Cohort's inclusion and exclusion criteria
**@neurIST_EMC_NL**	Aneurysmal SAH oriented cohort	Patients were recruited based on the diagnosis of IA by angiographic appearance (3D-DSA, 3D-MRA, or 3D-CTA) and by surgical documentation. SAH was verified by CT/MRI or by lumbar puncture. Patient had to be older than 14 years and had to provide consent. Patients with known genetic syndromes predisposing to IA (e.g., polycystic kidney disease or Ehlers-Danlos syndrome Type IV) were excluded from the study.
**@neurIST_GVA_CH**	Population-based cohort	
**@neurIST_HBAR_ES**	Aneurysmal SAH oriented cohort	
**@neurIST_MUP_HU**	Family-oriented IA cohort	
**@neurIST_UOXF_UK**	Aneurysmal SAH oriented cohort	
**@neurIST_USFD_UK**	Aneurysmal SAH oriented cohort	
**ACROSS_AUS**		Australasian Cooperative Research on Subarachnoid Hemorrhage cohort. None of the patients could be included due to lack of phenotypic patient and aneurysm location and size information.
**BASICMAR_ES**	Aneurysmal SAH oriented cohort	Since 2007, all consecutive patients with aSAH confirmed by cerebral angiography who were admitted and treated in Hospital del Mar in Barcelona, Spain were considered for inclusion in the study. Exclusion criteria were nonaneurysmal SAH, SAH due to another cerebral vascular malformation, lack of angiography data, or absence of genetic samples.
**CCC_USA**		Cincinnati control cohort. Data were used to verify the reference population.
**FIA1_USA and FIA2_USA**	Family-oriented IA cohort	Families with at least 2 members who had IA were ascertained through 26 clinical centers (41 sites) in North America, New Zealand, and Australia. Exclusion criteria included (i) fusiform-shaped unruptured IA of a major intracranial trunk artery; (ii) IA that is part of an arteriovenous malformation; (iii) family or personal history of polycystic kidney disease, Ehlers Danlos syndrome, Marfan's syndrome, fibromuscular dysplasia, or Moya-Moya disease; or (iv) failure to obtain informed consent from the patient or family members.
**FRCAN_CA**	Population-based cohort	Participants were recruited in Montréal and Québec City, Canada. Diagnoses were confirmed either by magnetic resonance angiography or by surgical confirmation (clipped or coiled).
**GERFHS1_USA and GERFHS2_USA**	Aneurysmal SAH oriented cohort	Patients in three counties in southwest Ohio and two counties in northern Kentucky (greater Cincinnati, Ohio region) whose SAH was confirmed by imaging and who consented to a detailed interview were included.
**GOSH_UK**	Aneurysmal SAH oriented cohort	Multicentre UK-wide study involving 20 neurosurgical centres. Included patients with proven IA, including angiographically-proven ruptured IA (irrespective of severity) and unruptured IA. Consent from subject(s) or nominated consultee(s) was obtained for all participants. Excluded patients with known inherited connective tissue disorders, such as Marfan's, Ehlers-Danlos syndrome, and adult polycystic kidney disease, as well as those with non-aneurysmal SAH (e.g., from arterio-venous malformations, trauma, mycotic aneurysms, and peri-mesencephalic SAH where no aneurysm was detected).

COHORTS	Cohort basis recruitment	Cohort's inclusion and exclusion criteria
**GSA_NL**	Aneurysmal SAH oriented cohort	Diagnosis of an IA was made either with computerized tomography angiogram, magnetic resonance angiogram, or cerebral digital subtraction angiogram; and was confirmed at surgery when applicable. Rupture of an aneurysm was defined by identification of acute subarachnoid or intracranial hemorrhage (through computerized tomography or magnetic resonance imaging) from a proven aneurysm. Subjects with SAH without saccular IA, non-saccular IA (such as fusiform aneurysms), and those with known genetic syndromes that are believed to predispose to IA (e.g., polycystic kidney disease or Ehlers-Danlos syndrome Type IV) were excluded from the study.
**HUCH_FI**		Rupture of an aneurysm was defined by identification of acute subarachnoid or intracranial hemorrhage (through computerized tomography or magnetic resonance imaging) from a proven aneurysm. Subjects with subarachnoid hemorrhage without saccular IA, non-saccular IA (such as fusiform aneurysms) and those with known genetic syndromes that are believed to predispose to IA (e.g., polycystic kidney disease or Ehlers-Danlos syndrome Type IV) were excluded from the study.
**ICAN_FR**	Population-based cohort	Sporadic and familial IA patients were collected. Exclusion criteria were fusiform or dissected IA, or IA related to an arteriovenous malformation. Patients were excluded if they had a family history of ADPKD, ED, Marfan's syndrome, fibromuscular dysplasia, or Moyamoya disease.
**JUMC_PL**	Aneurysmal SAH oriented cohort	IA cases were recruited from patients of the Department of Neurology and the Department of Neurosurgery and Neurotraumatology of the Jagiellonian University in Krakow, Poland. Subjects with ruptured or unruptured IA were recruited. Presence of IA was confirmed by intra-arterial angiogram, CTA, MRA, or intraoperatively.
**KIAD_FI**		Rupture of an aneurysm was defined by identification of acute subarachnoid or intracranial hemorrhage (through computerized tomography or magnetic resonance imaging) from a proven aneurysm. Subjects with subarachnoid hemorrhage without saccular IA, non-saccular IA (such as fusiform aneurysms) and those with known genetic syndromes that are believed to predispose to IA (e.g., polycystic kidney disease or Ehlers-Danlos syndrome Type IV) were excluded from the study.
**UCSF_USA**	Aneurysmal SAH oriented cohort	The University of California, San Francisco recruited a prospective cohort of adult patients with spontaneous SAH due to IA who were admitted to a tertiary-care referral center in San Francisco during 2003 to 2008. Cases were confirmed by non-contrast CT and cerebral angiogram. Exclusion criteria were the same as for the FIA cohort.
**UMCU2_NL**	Aneurysmal SAH oriented cohort	Patients diagnosed with an unruptured IA or aneurysmal SAH after 2011 in the University Medical Center Utrecht, The Netherlands, were recruited. Inclusion and exclusion criteria are identical to those of the GSA_NL cohort.

Table S3: Categories and definitions of the descriptors used to characterize patients and intracranial aneurysms

Data were collected prior to definitions established by the Common Data Elements project supported by the National Institute of Health.
Each raw data response was mapped to the appropriate element and standardized to the Common Data Elements definitions.

Descriptors	Categories	Definitions
**Basis of recruitment**	Subarachnoid hemorrhage	Patient diagnosed with subarachnoid bleed as a consequence of intracranial aneurysm rupture
	Symptomatic intracranial aneurysm	Patient diagnosed initially with a symptom associated with an intracranial aneurysm
	Incidental intracranial aneurysm	Patient incidentally diagnosed with intracranial aneurysm
	Case	Patients diagnosed with intracranial aneurysm(s) with unknown status of rupture
**Sex**	Female	Self-reported sex of the patient. Phenotype female
	Male	Self-reported sex of the patient. Phenotype male
**Family History of IA**	Yes	One or more 1st-degree relative(s) with intracranial aneurysm
	No	No 1st-degree relative with intracranial aneurysm
	Probably	A relative had a stroke but there is no definite diagnosis on the type of stroke
	Unknown	Unknown familial history of intracranial aneurysm
**Hypertension status**	AnyType	Yes – Untreated blood pressure greater than 140/90 mm Hg, and the patient does not take any antihypertensive treatment<br>Yes – Treated and controlled blood pressure greater than 140/90 mm Hg, the patient takes antihypertensive treatment, and the blood pressure is in normal range<br>Yes – Treated and uncontrolled blood pressure greater than 140/90 mm Hg, the patient takes antihypertensive treatment, but the blood pressure stays higher than normal blood pressure values
	Never	No – Blood pressure less than 120/80 mm Hg, or patient reports never having been diagnosed with high blood pressure
	Unknown	Hypertension status unknown
**Smoking status**	Current	Smoked (more than 300 cigarettes) and has smoked cigarettes with 6 months prior to recruitment
	Former	Smoked (more than 300 cigarettes), but stopped smoking at least 6 months ago
	No	Never smoked, or smoked fewer than 300 cigarettes in lifetime
	Unknown	Smoking status unknown
**Age at time of aneurysm rupture**	Age in years	Age of patient when intracranial aneurysm ruptured

Descriptors	Categories	Definitions
**Multiplicity status**	N number	Number of diagnosed intracranial aneurysms. In case of multiple aneurysms, the number of aneurysms was recorded if this information was available.
	Unknown	Unknown number of aneurysm
	Multiple aneurysm	Yes / No. In case of multiple aneurysms, the ruptured or the most critical aneurysm was identified by the recruiting investigator for the dataset.
**Ruptured status**	Yes	Patient known to have a ruptured intracranial aneurysm
	No	Patient known to never have a ruptured aneurysm
	Unknown	Rupture status of the aneurysm unknown
**Maximum aneurysm diameter at rupture**	Decimal	Maximum diameter size in mm
	Unknown	Maximal diameter unknown
**Aneurysm location**	*Abbreviation used in the manuscript*	*Location names given in the different cohorts*
	Acom	Anterior communicating artery, Acomm, ACoA, ACOM, Anterior circulation others, Comm anterior CoA forward, Comm anterior CoA forward-upward, Comm anterior CoA upward, Comm anterior CoA backward, Comm anterior CoA down
	A2	Distally to the Acom, Pericallosal cerebral artery, A2 segment ant, ACA-Anterior Cerebral Artery, A1-A2, PericalA, Pericallosal proximal, Pericallosal typical
	MCA	Middle cerebral artery, Sylvian bifurcation, M1 segment middle cerebral artery, M1 perforator artery, Middle cerebral bifurcation, Middle cerebral MCA main trunk
	Pcom	Posterior communicating artery starting proximal to the implantation of the posterior communication artery and extending up to the anterior choroidal artery, Posterior Comm, Pcomm, PcoA, PCOM
	ICA	Internal carotid artery extending from the anterior choroidal artery up to the ICA bifurcation including it, Anterior and superior wall carotid, Carotid bifurcation, Ant Choroidal segment carotid, other location carotid artery, Superior wall ICA, Lateral wall ICA CoP, Lateral wall ICA ChA
	cav-ICA	Intracavernous portion of ICA, Cavernous ICA
	ophtI-ICA	Ophthalmic segment of ICA starting immediately proximal to the ophthalmic artery departure and ending immediately proximal to the implantation of the posterior communication artery,

		Medial wall carotid, Ophthalmic Artery, OphtA, AOA, OA, ICA-ophth, Medial wall ICA ophthalmic, Medial wall ICA distal, Inferior wall ICA
	A1	A1 anterior segment: aneurysms located on the anterior cerebral artery distal to the ICA bifurcation but proximal to the anterior communicating artery
	Basilar	Basilar artery, Basilar Tip, basilar artery bifurcation, BA, P1 posterior cerebral
	VB	Vertebro-basilar artery, vertebro-basilar junction, V4 segment vertebral artery, Basilar trunk, AICA, Superior cerebellar artery, PICA, vertebral artery, VB-Other Basilar and Vertebral, VA, SCA, Vertebral trunk, Basilar others, Basilar SCA, Vertebral = PICA origin, PICA distal
	PCA	Posterior cerebral artery, P1 Posterior cerebral artery, P2 posterior cerebral artery, P1-P2 junction posterior cerebral artery. Aneurysms located distally to the basilar bifurcation.
	Other	Distal to sylvian bifurcation, Distal ant cerebral artery, Distal posterior cerebral artery, other location, Middle cerebral peripheral, Pericallosal distal, P3 posterior cerebral distal
	Infundibulum	Vessel calibre irregularities that could not be clearly identified as aneurysms
	Unknown	Unknown

Table S4: Number of patients with ruptured intracranial aneurysm enrolled in the study following coherent recruitment and known location of their intracranial aneurysm

COHORTS	Number of patients with IA	% of patients in the global cohort	Number of patients with ruptured IAs, N (%)
@neurIST_EMC_NL	53	0.7	36 (67.9)
@neurIST_GVA_CH	266	3.3	146 (54.9)
@neurIST_HBAR_ES	89	1.1	70 (78.7)
@neurIST_MUP_HU	40	0.5	17 (42.5)
@neurIST_UOXF_UK	67	0.8	52 (77.6)
@neurIST_USFD_UK	33	0.4	24 (72.7)
BASICMAR_ES	98	1.2	98 (100)
FIA1_USA	360	4.5	158 (43.9)
FIA2_USA	675	8.4	290 (43.0)
FRCAN_CA	9	0.1	7 (77.8)
GERFHS1_USA	12	0.2	12 (100)
GERFHS2_USA	32	0.4	32 (100)
GOSH_UK	2275	28.5	1679 (73.8)
GSA_NL	945	11.8	653 (69.1)
HUCH_FI	496	6.2	366 (73.8)
ICAN_FR	898	11.2	354 (39.4)
JUMC_PL	497	6.2	423 (85.1)
KIAD_FI	264	3.3	189 (71.6)
UCSF_USA	127	1.6	127 (100)
UMCU2_NL	756	9.5	701 (92.7)
TOTAL	7992	-	5434 (68.0)

aSAH-oriented cohorts: @neurIST_EMC_NL, @neurIST_HBAR_ES, @neurIST_UOXF_UK, @neurIST_USFD_UK, BASICMAR_ES, GERFHS1_USA, GERFHS2_USA, GOSH_UK, GSA_NL, JUMC_PL, UCSF_USA, and UMCU2_NL.

Family-oriented IA cohorts: @neurIST_MUP_HU, FIA1_USA, FIA2_USA, HUCH_FI, and KIAD_FI.

Population-based cohorts that recruited IA, aSAH, and familial cases: @neurIST_GVA_CH, FRCAN_CA, and ICAN_FR.

Table S5 – Consortium members

**The @neurIST project**	
Investigators	Alejandro Frangi (Universitat Pompeu Fabra. Barcelona, Spain)<br>Philippe Bijlenga (Hôpitaux Universitaire de Genève et Faculté de médecine de Genève. Geneva, Switzerland)<br>Martin Hofmann-Apitius (Fraunhofer Institut Algorithmen und Wissenschaftliches Rechnen. Sankt Augustin, Germany)<br>Rod Hose (Royal Hallamshire Hospital and University of Sheffield. United Kingdom)<br>Guy Lonsdale (NEC Laboratories Europe, IT Research Division. Sankt Augustin, Germany)<br>Antonio Arbona (NEC Laboratories Europe, IT Research Division. Sankt Augustin, Germany)<br>Peer Hasselmeyer (NEC Laboratories Europe, IT Research Division. Sankt Augustin, Germany)<br>Daniel Rüfenacht (Hôpitaux Universitaire de Genève et Faculté de médecine de Genève. Geneva, Switzerland).
Ethical and legal review committee	Jessica Wright (Durham University Department of Law. United Kingdom)<br>Jeremy Wilkins (Durham University Department of Law. United Kingdom)<br>Derick Beyleveld (Durham University Department of Law. United Kingdom)<br>Bernice Elger (Hôpitaux Universitaire de Genève et Faculté de médecine de Genève. Geneva, Switzerland).
Information platform development and maintenance	Alan Waterworth (Royal Hallamshire Hospital and University of Sheffield. United Kingdom)<br>Steven Wood (Royal Hallamshire Hospital and University of Sheffield. United Kingdom)<br>Jimison Iavindrasana (Hôpitaux Universitaire de Genève et Faculté de médecine de Genève. Geneva, Switzerland)<br>Rodolphe Meyer (Hôpitaux Universitaire de Genève et Faculté de médecine de Genève. Geneva, Switzerland)<br>Christoph Friedrich (Fraunhofer Institut Algorithmen und Wissenschaftliches Rechnen. Sankt Augustin, Germany)<br>Christian Ebeling (Fraunhofer Institut Algorithmen und Wissenschaftliches Rechnen. Sankt Augustin, Germany).
Epidemiology and statistics	Christian Ebeling (Fraunhofer Institut Algorithmen und Wissenschaftliches Rechnen. Sankt Augustin, Germany)<br>Philippe Bijlenga (Hôpitaux Universitaire de Genève et Faculté de médecine de Genève. Geneva, Switzerland)<br>Roelof Risselada (Erasmus University Medical Center. Rotterdam, The Netherlands)<br>Christoph Friedrich (Fraunhofer Institut Algorithmen und Wissenschaftliches Rechnen. Sankt Augustin, Germany)<br>Miriam CJM Sturkenboom (Erasmus University Medical Center. Rotterdam, The Netherlands).
Genotyping	Carole Proust, François Cambien (Unité Mixte de Recherche (UMR S937) and Pitié-Salpêtrière Post-Genomic Platform (P3S), Institut National de la Santé et de la Recherche Médicale (INSERM), University Pierre and Marie Curie (UPMC). Paris, France).
Participating Centers (In Order of	Hôpitaux Universitaire de Genève et Faculté de médecine de Genève. Geneva, Switzerland (Philippe Bijlenga, Max Jägersberg, Alister Rogers, Bawarjan Schatlo, Patrick Teta, Nathalie Isidor, Karl Schaller, Vitor Mendes-Pereira, Ana

Number of Recruited Patients):	Marcos Gonzalez, Ana Paula Narata, Zsolt Kulcsàr, Karl O Lovblad, Daniel A. Rüfenacht)<br>John Radcliffe Hospital, University of Oxford. Oxford, United Kingdom (Julia Yarnold, Paul Summers, Alison Clarke, Gulam Zilani, James Byrne)<br>Hospital Clinic. Barcelona, Spain (Juan Macho, Jordi Blasco)<br>University of Pècs Medical School. Pècs, Hungary (Peter Bukovics, Ferenc Kover, Istvan Hudak, Tamas Doczi)<br>Erasmus University Medical Center. Rotterdam, The Netherlands (Roelof Risselada, Miriam CJM Sturkenboom)<br>Royal Hallamshire Hospital. Sheffield, United Kingdom (Pankaj Singh, Alan Waterworth, Umang Patel, Stuart Coley, Patricia Lawford)<br>Hospital General de Catalunya. San Cugat del Valles, Spain (Teresa Sola, Elio Vivas).

The ICAN Study Group

Investigators	Hubert Desal, Romain Bourcier, Bertrand Isidor, Jérôme Connault, Pierre Lebranchu, Thierry Le Tourneau – University Hospital of Nantes. Nantes, France.<br>Chrisanthi Papagiannaki – University Hospital of Rouen. Rouen, France.<br>Michel Piotin, Hocine Redjem, Mikael Mazighi, Jean Philippe Desilles – Fondation Rotschild Hospital. Paris, France.<br>Olivier Naggara, Denis Trystram, Myriam Edjlali – Goujon,<br>Grégoire Boulouis, Christine Rodriguez, Waghi Ben Hassen – Saint Anne Hospital. Paris, France.<br>Suzanna Saleme, Charbel Mounayer, Aymeric Rouchaud – University Hospital of Limoges. Limoges, France.<br>Olivier Levrier, Pierre Aguettaz, Xavier Combaz – Clairval Hospital. Marseille, France.<br>Anne Pasco, Vincent l'Allinec – University Hospital of Angers. Angers, France.<br>Marc Bintner, Marc Molho, Pascale Gauthier – University Hospital of La Réunion. Saint-Denis de La Réunion, France.<br>Cyril Chivot – University Hospital of Amiens. Amiens, France.<br>Vincent Costalat, Cyril Darganzil, Alain Bonafé – University Hospital of Montpellier. Montpellier, France.<br>Anne Christine Januel, Caterina Michelozzi, Christophe Cognard, Fabrice Bonneville, Philippe Tall, Jean Darcourt – University Hospital of Toulouse. Toulouse, France.<br>Alessandra Biondi, Cristina Iosif – University Hospital of Besançon. Besançon, France.<br>Jean Christophe Ferre, Jean Yves Gauvrit, François Eugene, Hélène Raoult – University Hospital of Rennes. Rennes, France.<br>Jean Christophe Gentric, Julien Ognard – University Hospital of Brest. Brest, France.<br>René Anxionnat, Benjamin Gory, Serge Bracard, Anne Laure Derelle, Romain Tonnelet – University Hospital of Nancy. Nancy, France.<br>Laurent Spelle, Léon Ikka, Augustin Ozanne, Sophie Gallas, Jildaz Caroff, Nidal Ben Achour, Jacques Moret – Le Kremlin Bicêtre Hospital. Paris, France.<br>Emmanuel Chabert – University Hospital of Clermont-Ferrand. Clermont-Ferrand, France.<br>Jérôme Berge, Gaultier Marnat, Xavier Barreau, Florent Gariel – University Hospital of Bordeaux. Bordeaux, France.<br>Frédéric Clarencon, Eimad Shotar – La Pitié Salpétrière Hospital. Paris, France.<br>Mohammed Aggour – University Hospital of Saint Etienne. Saint Etienne, France.<br>Frédéric Ricolfi, Adrien Chavent, Pierre Thouant – University Hospital of Dijon. Dijon, France.<br>Pablo Lebidinsky, Brivael Lemogne – Colmar Hospital. Colmar, France.

	Denis Herbreteau, Richard Bibi, Kevin Janot – University Hospital of Tours. Tours, France.<br>Laurent Pierot, Sébastien Soize – University Hospital of Reims. Reims, France.<br>Marc Antoine Labeyrie, Christophe Vandendries, Emmanuel Houdart – Lariboisière Hospital. Paris, France.<br>Appoline Kazemi, Xavier Leclerc, Jean Pierre Pruvo, Nicolas Bricout – University Hospital of Lille. Lille, France.<br>Stéphane Velasco, Samy Boucebci – University Hospital of Poitiers. Poitiers, France.
<u>Epidemiology and statistics</u>	Christian Dina, Floriane Simonet, Eric Charpentier.
<u>Genotyping</u>	Richard Redon, Gervaise Loirand, Jean-Jacques Schott, Stéphanie Chatel, Emmanuelle Bourcereau, Estelle Baron, Stéphanie Bonnaud.

<u>Genetics and Observational Subarachnoid Haemorrhage (GOSH) Study investigators</u>

<u>Investigators</u>	Dr Stephen Bonner. The James Cook University Hospital. Middlesbrough, UK<br>Mr Daniel Walsh. King's College Hospital NHS Foundation Trust. London, UK.<br>Mr Diederik Bulters. University Hospital Southampton NHS Foundation Trust. Southampton, UK.<br>Mr Neil Kitchen. The National Hospital of Neurology and Neurosurgery. London, UK.<br>Prof Martin Brown. UCL Queen Square Institute of Neurology. London, UK.<br>Ms Joan Grieve. The National Hospital of Neurology and Neurosurgery. London, UK.<br>Mr Gareth Roberts. Royal Preston Hospital. UK.<br>Mr Timothy Jones. St George's Hospital. London, UK.<br>Mr Giles Critchley. Hurstwood Park Neurological Centre. UK.<br>Prof Pankaj Sharma. Imperial Healthcare, Charing Cross Hospital. London, UK.<br>Mr Richard Nelson. Frenchay Hospital. Bristol, UK.<br>Prof Peter Whitfield. Derriford Hospital. Plymouth, UK.<br>Mr Stuart Ross. Leeds General Infirmary. UK.<br>Mr Hiren Patel. Salford Royal Hospital. UK.<br>Mr Paul Eldridge. The Walton Centre. Liverpool, UK.<br>Dr Kari Saastamoinen. The Royal London Hospital. London, UK.<br>Mr Umang Patel. Sheffield Teaching Hospitals NHS Trust. UK.<br>Dr Enas Lawrance. Mayday Hospital. Croydon, UK.<br>Dr Subha Vandabona. Mount Gould Hospital. Plymouth, UK.<br>Professor David Mendelow. Newcastle University Hospitals NHS Trust.<br>Ms Rachel Teal. Oxford Radcliffe Infirmary. UK.<br>Dr Orlando Warner. Oxford Radcliffe Infirmary. UK.<br>Professor Peter Kirkpatrick. Addenbrooke's Hospital. Cambridge, UK

<u>International Stroke Genetics consortium (ISGC)</u>

Sudha Seshadri. Department of Neurology, Boston University School of Medicine, Framingham Heart Study. Boston, MA, USA<br>Laura Kilarski. University of Cologne. Germany<br>Hyacinth I Hyacinth. Aflac Cancer and Blood Disorder Center, Department of Pediatrics, Emory University School of Medicine. Atlanta, GA, USA<br>Jamary Oliveira-Filho. Stroke and Cardiomyopathy Clinics, Federal University of Bahia, Brazilian National Research Committee (CNPq), Instituto Nacional de Ciencia e Tecnologia em Doencas Tropicais (INCT-DT). Salvador, Brazil<br>Sandro Marini. Center for Human Genetic Research, Harvard Medical School. Boston, MA, USA<br>Sunaina Yadav. Imperial College Cerebrovascular Research Unit, Department of Clinical Neuroscience, Imperial College London. London, UK

Paul Nyquist. Department of Anesthesiology and Critical Care Medicine, Johns Hopkins Medicine. Baltimore, MD, USA
Cathryn Lewis. Department of Medical and Molecular Genetics, King's College London, SGDP Centre, Institute of Psychiatry, Psychology & Neuroscience, King's College London. London, UK
Bjorn Hansen. Department of Clinical Sciences Lund, Neurology, Lund University, Department of Neurology and Rehabilitation Medicine, Skåne University Hospital. Lund, Sweden
Bo Norrving. Sahlgrenska Academy at University of Gothenburg, Lund University, Department of Neurology and Rehabilitation Medicine, Skåne University Hospital. Lund, Sweden
Gustav Smith. Department of Clinical Sciences Lund, Cardiology, Lund University, The Section for Heart Failure and Valvular Disease, VO Heart and Lung medicine, Skane University Hospital. Lund, Sweden
Jonathan Rosand. Department of Neurology, Massachusetts General Hospital, Center for Human Genetic Research. Massachusetts General Hospital (MGH). Boston, MA, USA
Alessandro Biffi. Division of Behavioral Neurology, Department of Neurology, Massachusetts General Hospital, Center for Human Genetic Research, Massachusetts General Hospital (MGH). Boston, MA, USA
Christina Kourkoulis. Department of Neurology, Massachusetts General Hospital, Center for Human Genetic Research, Massachusetts General Hospital (MGH). Boston, MA, USA
Chris Anderson. Center for Genomic Medicine, Massachusetts General Hospital (MGH), J. Philip Kistler Stroke Research Center, Department of Neurology, Massachusetts General Hospital (MGH). Boston, MA, USA
Anne-Katrin Giese. Department of Neurology, Massachusetts General Hospital, Harvard Medical School, Department of Neurology, J. Philip Kistler Stroke Research Center, MGH. Boston, MA, USA
Ralph Sacco. Department of Neurology, Miller School of Medicine, University of Miami. Miami, FL, USA
Pankaj Sharma. Institute of Cardiovascular Research, Royal Holloway University of London. London, UK
Oh Young Bang. Department of Neurology, Samsung Medical Center, Sungkyunkwan University School of Medicine. Seoul, Korea
Jong-Won Chung. Department of Neurology, Samsung Medical Center, Sungkyunkwan University School of Medicine. Seoul, Korea
Gyeong-Moon Kim. Department of Neurology, Samsung Medical Center, Sungkyunkwan University School of Medicine. Seoul, Korea
Qishuai Zhuang. Department of Pharmacology, School of Life Science and Biopharmaceutical Sciences, Shenyang Pharmaceutical University. Shenyang, China
Wayne Sheu. Department of Internal Medicine, Taichung Veterans General Hospital. Taichung, Taiwan
Steven Lubitz. Department of Cardiology, Massachusetts General Hospital, Harvard Medical School. Boston, MA, USA
David Werring, Stroke Research Centre, Department of Brain Repair and Rehabilitation, University College London Institute of Neurology. London, UK
John Hardy. Reta Lila Weston Institute, UCL Institute of Neurology, University College London. London, UK
June Smalley. Reta Lila Weston Institute, UCL Institute of Neurology, University College London. London, UK
Romain Bourcier. Department of Neuroradiology, University Hospital of Nantes. Nantes, France
Joanna Howson. MRC/BHF Cardiovascular Epidemiology Unit, Department of Public Health and Primary Care, University of Cambridge. Cambridge, UK
Alessandra Granata. Department of Clinical Neurosciences, University of Cambridge. Cambridge, UK
Anna Drazyk. Department of Clinical Neurosciences, University of Cambridge. Cambridge, UK
Hugh Markus. Stroke Research Group, Division of Clinical Neurosciences, University of Cambridge. Cambridge, UK
Joanna Wardlaw. Neuroimaging Sciences, University of Edinburgh, Centre for Clinical Brain Sciences, University of Edinburgh. Edinburgh, UK

Braxton Mitchell. Department of Medicine, University of Maryland School of Medicine, Geriatrics Research and Education Clinical Center, Baltimore Veterans Administration Medical Center. Baltimore, MD, USA
John Cole. Department of Neurology, University of Maryland School of Medicine and Baltimore VAMC. Baltimore, MD, USA
Anbupalam Thalamuthu. Centre for Healthy Brain Ageing, Psychiatry, University of New South Wales (UNSW). Sydney, NSW, Australia
Jemma Hopewell. Clinical Trial Service Unit and Epidemiological Studies Unit, Nuffield Department of Population Health, University of Oxford. Oxford, UK
Robin Walters, Clinical Trial Service Unit and Epidemiological Studies Unit, Nuffield Department of Population Health, University of Oxford. Oxford, UK
Iain Turnbull. Clinical Trial Service Unit and Epidemiological Studies Unit, Nuffield Department of Population Health, University of Oxford. Oxford, UK
Bradford Worrall. Departments of Neurology and Public Health Sciences, University of Virginia School of Medicine. Charlottesville, VA, USA
Josh Bis. Cardiovascular Health Research Unit, Department of Medicine, University of Washington. Seattle, WA, USA
David Tirschwell. Comprehensive Stroke Care at the UW Medicine Stroke Center at Harborview Medical Center, University of Washington. Seattle, WA, USA
Alex Reiner. Department of Epidemiology, University of Washington, Fred Hutchinson Cancer Research Center, University of Washington. Seattle, WA, USA
Raj Dhar. Department of Neurology, Washington University School of Medicine. St. Louis, MO, USA
Laura Heitsch. Department of Neurology, Radiology, and Biomedical Engineering, Washington University School of Medicine. St. Louis, MO, USA
Jin-Moo Lee. Department of Neurology, Radiology, and Biomedical Engineering, Washington University School of Medicine. St. Louis, MO, USA
Janne Mortenson. Department of Neurology, Aarhus University Hospital. Aarhus, Denmark
Sylvia Wassertheil-Smoller. Department of Epidemiology and Population Health, Albert Einstein College of Medicine. New York, NY, USA
Kameshwar Prasad. Department of Neurology and Stroke Unit, Director of Clinical Epidemiology, All India Institute of Medical Sciences (AIIMS). New Delhi, India
Mark Fisher. Department of Neurology, University of Massachusetts Medical School. Worchester, MA, USA
Christopher Traenka. Neurology Clinic, Basel University Hospital. Basel, Switzerland
Xingwu Wang. Beijing Hypertension League Institute. Beijing, China
Yongjun Wang. Center of Stroke, Beijing Institute for Brain Disorders, Department of Neurology, Beijing Tiantan Hospital, Capital Medical University. Beijing, China
Francois Rouanet. Department of Neurology, Bordeaux University Hospital. Bordeaux, France
Igor Sibon. Department of Neurology, Bordeaux University Hospital. Bordeaux, France
Chloé Sarnowski. Department of Biostatistics, Boston University. Boston, MA, USA
Pauline Maillard. Department of Neurology, University of California at Davis. Davis, CA, USA
Aleksandra Pikula. Department of Neurology, University of Toronto. Toronto, Ontario, Canada
Hugo Javier Aparicio. Department of Neurology, Boston University School of Medicine. Boston, MA, USA
Philip Wolf. Boston University School of Medicine. Boston, MA, USA
Josee Dupuis. Boston University School of Public Health. Boston, MA, USA
Qiong Yang. Boston University School of Public Health. Boston, MA, USA
Gustavo Luvizutto. Rehabilitation department, Botucatu Medical School, Botucatu School of Medicine, University Estadual Paulista Júlio de Mesquita Filho, District of Rubião Junior. Botucatu, Brazil
Daniel Chasman. Division of Preventive Medicine, Brigham and Women's Hospital, Harvard Medical School. Boston, MA, USA
Kathryn Rexrode. Department of Medicine, Brigham and Women's Hospital, Harvard Medical School. Boston, MA, USA

Andrea Harriot. Department of Neurology, Brigham and Women's Hospital, Massachusetts General Hospital. Boston, MA, USA
Chia-Ling Phuah. Center for Human Genetic Research, Massachusetts General Hospital (MGH), Division of Neurocritical Care and Emergency Neurology, Department of Neurology. Boston, MA, USA
Gustavo Santo. Stroke Unit, Centro Hospitalar e Universitário de Coimbra, CHUC. Coimbra, Portugal
Ales Tomek. Department of Neurology, 2nd Faculty of Medicine, Charles University in Prague and Motol University Hospital. Prague, Czech Republic
Jen Gerard. Department of Cardiology, Children's Hospital of Wisconsin. Milwaukee, WI, USA
Cara Carty. Children's Research Institute, Children's National Medical Center, Center for Translational Science, George Washington University. Washington, DC, USA
Guiyou Liu. Chinese Academy of Sciences (CAS). Beijing, China
Sanjith Aaron. Department of Neurological Sciences, Christian Medical College. Vellore, India
Christhunesa S. Christudass. Department of Neurological Sciences, Christian Medical College. Vellore, India
BSB Salomi. Department of Neurological Sciences, Christian Medical College. Vellore, India
Dharambir Sanghera. Department of Pediatrics, College of Medicine, University of Oklahoma Health Sciences Center, Department of Pharmaceutical Sciences, College of Pharmacy, University of Oklahoma Health Sciences Center. Oklahoma City, OK, USA
Amelia Boehme. Department of Neurology, Columbia University. New York, NY, USA
Mitchell Elkind. Neurological Institute of New York, Columbia University College. New York, NY, USA
Gudmar Thorleifsson. DeCODE genetics/AMGEN inc. Reykjavik, Iceland
Solveig Gretarsdottir. DeCODE genetics/AMGEN inc. Reykjavik, Iceland
Leslie Lange. University of Colorado Denver – Anschutz Medical Campus. Denver, CO, USA
Natalia Rost. Department of Neurology, Massachusetts General Hospital, Harvard Medical School, Department of Neurology, J. Philip Kistler Stroke Research Center, MGH. Boston, MA, USA
Michael James. Translational Acute Brain Injury Research Center, Duke University. Durham, NC, USA
Jill Stewart. Department of Neurology, Duke University School of Medicine. Durham, NC, USA
Larry Goldstein. Department of Medicine, Duke Stroke Center, Duke University School of Medicine. Durham, NC, USA
Salina Waddy. Cardiovascular and Stroke Genetics Working Group. Emory University, Atlanta, GA, USA
Carla Ibrahim-Verbaas. Department of Neurology, Erasmus MC. Rotterdam, Netherlands
Dina Vojinovic. Department of Epidemiology, Erasmus MC, University Medical Center Rotterdam, Clinic for Neurology and Psychiatry for Children and Youth, University of Belgrade, Faculty of Medicine. Rotterdam, Netherlands
Arfan Ikram. Department of Epidemiology, Erasmus University Medical Center. Rotterdam, Netherlands
Hieab Adams. Department of Epidemiology, Erasmus University Medical Center. Rotterdam, Netherlands
Fadi Charchar. L.E.W. Carty Cardiovascular Genomics laboratory, Federation University Australia. Ballarat, Australia
Vincent Thijs. Stroke Division, Florey Institute of Neuroscience and Mental Health, Department of Neurology, Austin Health. Heidelberg, Australia
Eugenio Parati. Laboratory of Cellular Neurobiology, Fondazione IRCCS Istituto Neurologico C. Besta, Cerebrovascular Disease Unit, Cerebrovascular Disease Unit, Fondazione IRCCS Istituto Neurologico C. Besta. Milan, Italy
Giorgio Boncoraglio. Department of Cerebrovascular Diseases, Fondazione IRCCS Istituto Neurologico Carlo Besta. Milan, Italy
Charles Kooperberg. Division of Public Health Sciences, Fred Hutchinson Cancer Research Center. Seattle, WA. USA
Sherrine Abboud, Laboratory of Experimental Neurology, Free University of Brussels (ULB). Brussels, Belgium
Ramin Zand. Department of Neuroscience, Geisinger Health System. Danville, PA, USA

Philippe Bijlenga. Departement des neurosciences cliniques, Clinique de neurochirurgie, Geneva University Hospital. Geneva, Switzerland
Magdy Selim. Beth Israel Deaconess Medical Center, Harvard Medical School. Boston, MA, USA
Caspar Grond-Ginsbach. Department of Neurology, Heidelberg University Hospital. Heidelberg, Germany
Olli Happola. Department of Neurology, Helsinki University Central Hospital. Helsinki, Finland
Daniel Strbian. Department of Neurology, Helsinki University Central Hospital, Clinical Neurosciences, Neurology, University of Helsinki. Helsinki, Finland
Liisa Tomppo. Department of Neurology, Helsinki University Central Hospital. Helsinki, Finland
Hanne Sallinen. Department of Neurology, Helsinki University Hospital. Helsinki, Finland
Abhishek Pathak. Department of Neurology, Heritage Hospital, Department of Neurology, Neuro Expert Clinic. Varanasi, India
Dorothea Pfeiffer. Department of Neurology, Heidelberg University Hospital. Heidelberg, Germany
Pablo Bonardo. Department of Neurology, Hospital Británico de Buenos Aires. Ciudad Autónoma de Buenos Aires, Argentina
Joao Jose Freitas de Carvalho. Hospital Geral de Fortaleza/SUS Fortaleza. Ceará, Brazil
Priscila Ribeiro. Hospital Governador Celso Ramos. Florianópolis, Brazil
Nuria Torres. Stroke Pharmacogenomics and Genetics, Hospital Mútua de Terrassa, Fundació Docència i Recerca Mútua Terrassa. Terrassa, Spain
Miguel Barboza. Department of Neurology, Hospital Rafael A. Calderon Guardia. San Jose, Costa Rica
Melanie Laarman. Cardiac Development and Genetics Group, Hubrecht Institute. Utrecht, Netherlands
Roberta Carriero. Inflammation and immunology laboratory, Humanitas Clinical Institute. Pavia, Italy
Elizabeth Holliday. Public Health Stream, Hunter Medical Research Institute, Faculty of Health and Medicine, University of Newcastle. New Lambton, Australia
Androniki Plomaritoglou. Neurology, Hygeia Hospital. Marousi, Greece
Johan Bjorkegren. Department of Genetic and Genomic Sciences, Icahn School of Medicine. New York, NY, USA
Yu-Feng Yvonne Chan. Department of Genetic and Genomic Sciences, Icahn School of Medicine, Mount Sinai. New York, NY, USA
Villi Gudnason. Icelandic Heart Association Research Institute, Faculty of Medicine, University of Iceland. Kopavogur, Iceland
Jordi Jimenez-Conde. Neurovascular Research Group (NEUVAS). Neurology Department, IMIM–Hospital del Mar. Barcelona, Spain
Carolina Soriano. Department of Neurology, Neurovascular Research Group, IMIM–Hospital del Mar. Barcelona, Spain
Jaume Roquer. Neurology Service, IMIM–Hospital del Mar, Institut Hospital del Mar d'investigacions Mèdiques. Barcelona, Spain
Paul Bentley. Faculty of Medicine, Department of Medicine, Imperial College London. London, UK
Dipender Gill. Centre for Pharmacology and Therapeutics, Imperial College London. London, UK
Ganesh Chauhan. Centre for Brain Research, Indian Institute of Science. Bangalore, India
Elisabeth Tournier-Lasserve. UMR-S 740, INSERM. Paris, France
Sara Kaffashian. INSERM U1219 Bordeaux Population Health Research Center. Bordeaux, France
Cecilia Samieri. INSERM U1219 Bordeaux Population Health Research Center, ISPED, University of Bordeaux. Bordeaux, France
Carole Dufouil. INSERM U1219 Bordeaux Population Health Research Center, Department of Public Health, CHU Bordeaux. Bordeaux, France
Stephanie Debette. INSERM U1219 Bordeaux Population Health Research Center, Bordeaux, France, University of Bordeaux, France
Aniket Mishra, INSERM U1219 Bordeaux Population Health Research Center, Bordeaux, France, University of Bordeaux, France
Lawrence Wee. Data Analytics Department, Institute for Infocomm Research (I2R). Singapore, Singapore
Saima Siddiqi. Institute of Biomedical and Genetic Engineering. Islamabad, Pakistan

Jer-Yuarn Wu. Institute of Biomedical Sciences, Academia Sinica. Taipei, Taiwan
Tai-Ming Ko. Institute of Biomedical Sciences, Academia Sinica, Graduate Institute of Integrated Medicine, China Medical University. Taipei, Taiwan
Silvia Bione. Computational Biology Unit, Institute of Molecular Genetics–National Research Council. Pavia, Italy
Katarina Jood. Department of Clinical Neuroscience, Institute of Neuroscience and Physiology, Sahlgrenska Academy at University of Gothenburg. Gothenburg, Sweden
Turgut Tatlisumak. Department of Clinical Neuroscience, Institute of Neuroscience and Physiology, Sahlgrenska Academy at University of Gothenburg, Sahlgrenska University Hospital. Gothenburg, Sweden
Lukas Holmegaard. Department of Clinical Neuroscience, Institute of Neuroscience and Physiology, Sahlgrenska Academy at University of Gothenburg. Gothenburg, Sweden
Antonio Arauz. Stroke Clinic, Instituto Nacional de Neurologia y Neurocirugia, Manuel Velasco Suarez. Mexico City, Mexico
Michal Korostynski. Department of Molecular Neuropharmacology, Polish Academy of Sciences. Warsaw, Poland
Lenore Launer. Laboratory of Epidemiology and Population Science, Intramural Research Program, National Institutes of Health, National Institute on Aging. Bethesda, MD, USA
Suo Yue. Ion Torrent by Life Technologies (company), Carnegie Mellon University. San Francisco, CA, USA
Anna Bersano. Cerebrovascular Unit, IRCCS Foundation C. Besta Neurological Institute. Milan, Italy
Tomasz Dziedzic. Department of Neurology, Jagiellonian University. Krakow, Poland
Karol Józef Juchniewicz. Jagiellonian University. Krakow, Poland
Adamski Mateusz. Jagiellonian Centre for Experimental Therapeutics, Jagiellonian University. Krakow, Poland
Joanna Pera. Department of Neurology, Jagiellonian University Medical College. Krakow, Poland
Agnieszka Slowik. Department of Neurology, Jagiellonian University Medical College. Krakow, Poland
Andrzej Urbanik. Department of Radiology, Jagiellonian University Medical College. Krakow, Poland
Jerzy Gasowski. Department of Internal Medicine and Gerontology, Jagiellonian University Medical College. Krakow, Poland
Rafał Olszanecki. Faculty of Medicine, Jagiellonian University Medical College. Krakow, Poland
Marcin Wnuk. Jagiellonian University Medical College. Krakow, Poland
Christopher Levi. John Hunter Hospital, Hunter Medical Research Institute and University of Newcastle. Newcastle, Australia
Aaron Gusdon. Department of Neurology, Johns Hopkins Medicine, University of Pittsburgh School of Medicine. Baltimore, MD, USA
Konstantinos Kostulas. Department of Clinical Neuroscience, Karolinska Institute. Stockholm, Sweden
Milita Crisby. Division of Clinical Geriatrics, Neurobiology Care Sciences and Society, Karolinska Institute. Huddinge, Sweden
Lina Keller. Karolinska Institutet. Stockholm, Sweden
Kristina Schlicht. Institut für Medizinische Informatik und Statistik, Kiel University. Kiel, Germany
Jessye Maxwell. Social genetic and Developmental Psychiatry, King's College London. London, UK
Christof Haffner. Institute for Stroke and Dementia Research, Klinikum der Universität München. Munich, Germany
Marco Duering. Institute for Stroke and Dementia Research, Klinikum der Universität München, Ludwig-Maximilians-University LMU. Munich, Germany
Jeremiasz Jagiella. Krakow University Hospital. Krakow, Poland
Robin Lemmens. Department of Neurosciences, Experimental Neurology, KU Leuven – University of Leuven, Department of Neurology, University Hospitals Leuven, VIB Center for Brain & Disease Research. Leuven, Belgium
Jun Hata. Department of Epidemiology and Public Health, Kyushu University, Graduate School of Medical Sciences. Fukuoka, Japan

Toshiharu Ninomiya. Department of Epidemiology and Public Health, Kyushu University, Graduate School of Medical Sciences. Fukuoka, Japan
Vinh Nguyen. School of Psychological Science, La Trobe University. Melbourne, Australia
Bjorn Logi Thorarinsson. Department of Neurology, Landspítali Háskólasjúkrahús. Reykjavík, Iceland
Saskia Lesnik Oberstein. Department of Clinical Genetics, Leiden University Medical Center. Leiden, Netherlands
Tsong-Hai Lee. Neurology department and Stroke center, Linkou Chang Gung Memorial Hospital. Taipei, Taiwan
Alexandr Rakitko. Lomonosov Moscow State University. Moscow, Russia
Rainer Malik. Institute for Stroke and Dementia Research, Ludwig-Maximilians-University, Klinikum der Universität München. Munich, Germany
Martin Dichgans. Institute for Stroke and Dementia Research, Ludwig-Maximilians-University, Klinikum der Universität München, Munich Cluster for Systems Neurology (SyNergy). Munich, Germany
Arne Lindgren. Department of Clinical Sciences Lund, Neurology, Lund University, Department of Neurology and Rehabilitation Medicine, Skåne University Hospital. Lund, Sweden
Johan Wasselius. Department of Clinical Sciences Malmö, Lund University, Vascular Center, Skåne University Hospital. Malmö, Sweden
Mattias Drake. Diagnostic Radiology, Lund University. Lund, Sweden
Olle Melander. Department of Clinical Sciences, Lund University. Lund, Sweden
Martin Stenman. Geriatric Medicine, Lund University. Lund, Sweden
Andreea Ilinca. Neurology, Lund University. Lund, Sweden
Julie Staals. Department of Neurology, Maastricht University Medical Center (MUMC+), Cardiovascular Research Institute Maastricht (CARIM). Maastricht, Netherlands
Ariane Sadr-Nabavi. Medical Genetics Research Center (MGRC), Mashhad University of Medical Sciences (MUMS), School of Medicine, Department of Medical Genetics, Mashhad University of Medical Sciences (MUMS), Faculty of Medicine. Mashhad, Iran
Katherine Crawford. Center for Genomic Medicine, Massachusetts General Hospital. Boston, MA, USA
Jose Florez. Center for Human Genetic Research, MGH, Massachusetts General Hospital, Harvard Medical School. Boston, MA, USA
Umme Lena. Massachusetts General Hospital, South Hadley, MA, USA
Farrah Mateen. Department of Neurology, Massachusetts General Hospital. Boston, MA, USA
Hakan Ay. Department of Neurology, Massachusetts General Hospital. Boston, MA, USA
Ona Wu. Department of Neurology, JPK Stroke Research Center, Massachusetts General Hospital and Harvard Medical School, Department of Radiology, Athinoula A. Martinos Center for Biomedical Imaging, MGH. Boston, MA, USA
Markus Schirmer. Department of Neurology, J. Philip Kistler Stroke Research Centre, Massachusetts General Hospital and Harvard Medical School. Boston, MA, USA
Javier Romero. Division of Neurocritical Care and Emergency Neurology, Department of Neurology, Massachusetts General Hospital, Harvard Medical School, J. P. Kistler Stroke Research Center, Massachusetts General Hospital, Harvard Medical School. Boston, MA, USA
Steve Cramer. Neurology, Anatomy & Neurobiology, and Physical Medicine & Rehabilitation, University of California Irvine. Irvine, CA, USA
Polina Golland. The Computer Science and Artificial Intelligence Laboratory, Massachusetts Institute of Technology. Cambridge, MA, USA
Bertram Mueller-Myhsok. Max-Planck-Institute of Psychiatry. Munich, Germany
Robert Brown. Department of Neurology, Mayo Clinic. Rochester, MN, USA
James Meschia. Department of Neurology, Mayo Clinic. Jacksonville, FL, USA
Owen A. Ross. Department of Medical Genetics and Neuroscience, Mayo Clinic. Jacksonville, FL, USA
Thomas Brott. Neurology, Mayo Clinic, Department of Surgery, University of Medicine and Dentistry of New Jersey. Jacksonville, FL, USA
Guillaume Pare. Population Health Research Institute, McMaster University. Hamilton, Ontario, Canada

Mike Chong. Population Health Research Institute, McMaster University. Hamilton, Ontario, Canada
Ossama Yassin Mansour. Department of Neurology, Medical School of Alexandria. Alexandria Governorate, Egypt
Bartosz Karaszewski. Department of Adult Neurology, Medical University of Gdansk & University Clinical Centre in Gdansk. Gdansk, Poland
Christian Enzinger. Department of Neurology, Medical University of Graz. Graz, Austria
Helena Schmidt. Institute of Molecular Biology and Biochemistry, Medical University of Graz. Graz, Austria
Reinhold Schmidt. Department of Neurology, Medical University of Graz. Graz, Austria
Stephan Seiler. Department of Neurology, Medical University of Graz. Graz, Austria
Alexander Pichler. Department of Neurology, Medical University of Graz. Graz, Austria
Bruce Ovbiagele. Department of Neurology, Medical University of South Carolina. Charleston, SC, USA
Yoshiji Yamada. Department of Human Genomics, Mie University, Life Science Research Center. Mie, Japan
Tatjana Rundek. Department of Neurology, Miller School of Medicine, University of Miami. Miami, FL, USA
Susan Blanton. John P. Hussman Institute for Human Genomics, Miller School of Medicine, University of Miami. Miami, FL, USA
Joseph Chern. Department of Neurology, College of Medicine, National Cheng Kung University. Tainan, Taiwan
Chris O'Donnell. Cardiovascular Epidemiology and Human Genomics Branch, National Heart, Lung and Blood Institute, Intramural Research, Veterans Administration Healthcare, Center for Population Genomics. Boston, MA, USA
Roderick Corriveau. National Institute of Neurological Disorders and Stroke, National Institute of Health, University of California. Bethesda, MD, USA
Pallab Bhattacharya. National Institute of Pharmaceutical Education and Research. Ahmedabad, India
Andrew Singleton. Laboratory of Neurogenetics, National Institute on Aging, Intramural Research Program. Bethesda, MD, USA
Katrina Gwinn. National Institute of Neurological Disorders and Stroke, National Institutes of Health. Bethesda, MD, USA
Bharatendu Chandra. General Neurology and Stroke, National University Hospital. Singapore, Singapore
Christopher Chen. Department of Pharmacology, National University of Singapore. Singapore, Singapore
Raj Kalaria. Institute of Neuroscience, Newcastle University. Newcastle, UK
Jim Koenig. Division of Neuroscience, Stroke Program in the Neural Environment, NINDS. Bethesda, MD, USA
Om Prakash Singh. Department of Environmental Studies, North Eastern Hill University (NEHU). Shillong, India
Akintomi Olugbodi. Obafemi Awolowo University. Ile-Ife, Nigeria
Bishwa Sapkota. Harold Hamm Diabetes Center, Oklahoma University. Oklahoma City, OK, USA
Rebecca Jackson. Center for women's health, Ohio State University Wexner Medical Center. Columbus, OH, USA
Eva Giralt. Parc de Salut Mar. Barcelona, Spain
Danish Saleheen. Department of Genetics, Perelman School of Medicine, University of Pennsylvania. Philadelphia, Pennsylvania, USA
Frank-Erik de Leeuw. Department of Neurology, Radboud University Medical Center. Nijmegen, Netherlands
Karin Klijn. Department of Neurology, Radboud University Medical Centre, Donders Institute for Brain, Cognition and Behaviour, Department of Neurology and Neurosurgery, University Medical Center, Brain Center Rudolf Magnus. Nijmegen, Netherlands
Jes Olesen. Department of Clinical Medicine, Rigshospitalet – Neurocentret. Glostrup, Denmark
Yoichiro Kamatani. Laboratory for Statistical Analysis, RIKEN Center for Integrative Medical Sciences, Center for Genomic Medicine, Kyoto University Graduate School of Medicine. Yokohama, Japan

Michiaki Kubo. RIKEN Center for Integrative Medical Sciences.Yokohama, Japan
Yukinori Okada. Laboratory for Statistical Analysis, RIKEN Center for Integrative Medical Sciences, Department of Statistical Genetics, Osaka University Graduate School of Medicine. Yokohama, Japan
David Spence. Stroke Prevention & Atherosclerosis Research Centre, Robarts Research. London, Ontario, Canada
Annie Pedersen. Västra Vötalandsregionen, Sahlgrenska Academy at University of Gothenburg. Gothenburg, Sweden
Maja Olsson. Department of Medical and Clinical Genetics, Institute of Biomedicine, Sahlgrenska Academy at University of Gothenburg. Gothenburg, Sweden
Turgut Tatlisumak. Department of Clinical Neurosciences/Neurology, Sahlgrenska Academy at University of Gothenburg, Institute of Neuroscience and Physiology, Sahlgrenska University Hospital. Gothenburg, Sweden
Giacomo Giacalone. Neurology Department, San Raffaele Scientific Institute, University Vita–Salute San Raffaele. Milan, Italy
Juan José Martín. Department of Neurology, Sanatorio Allende. Cordoba, Argentina
Rodrigo Bazan. Department of Neurology, São Paulo State University (Unesp) Botucatu Medical School. Botucatu, Brazil
Gabriel Braga. Hospital das Clínicas, São Paulo State University (Unesp) Botucatu Medical School. Botucatu, Brazil
Steve Bevan. School of Life Science, University of Lincoln. Lincoln, UK
Huichun Xu. Medicine department, School of Medicine, University of Maryland. Baltimore, MD, USA
Tim Assimes. Cardiovascular Medicine, School of Medicine, Stanford University. Stanford, CA, USA
Jamie Wright. Anderson Cancer Center, MD Anderson Clinical Cancer Genetics, School of Medicine, University of Texas. Houston, TX, USA
Anton Raskurazhev. Scientific Center of Neurology. Moscow, Russia
Wei Ling Lee. National Neuroscience Institute, Singapore General Hospital, Department of Pediatrics, National University of Singapore, Yong Loo Lin School of Medicine. Singapore, Singapore
Eng King Tan. Department of Neurology, Singapore General Hospital, National Neuroscience Institute, Neuroscience and Behavioral Disorders Program, Duke–National University of Singapore Graduate Medical School. Singapore, Singapore
Philippe Burri. Department of Cardiology, Skane University Hospital. Malmö, Sweden
Petrea Frid. Critical Care Neurology, Skåne University Hospital, Lund University. Malmö, Sweden
Christian Opherk. Department of Neurology, SLK-Kliniken Heilbronn GmbH. Heilbronn, Germany
Chaeyoung Lee. Department of Bioinformatics and Life Science, Soongsil University. Seoul, Korea
David Tregouet. Team Genomics and Pathophysiology of Cardiovascular Diseases, Sorbonne Universités, UPMC Univ. Paris 06, INSERM, UMR_S 1166, ICAN Institute for Cardiometabolism and Nutrition. Paris, France
Zhen Deng. Department of Neurology, Southern Medical University, FIMMU. Guangzhou, China
Hung Yi Chiou. School of Public Health, College of Public Health and Nutrition, Taipei Medical University. Taipei, Taiwan
Mahdi Habibi-Koolaee. Department of Health Information Management, Tehran University of Medical Sciences (TUMS). Tehran, Iran
Murali Vijayan. Garrison Institute on Aging, Texas Tech University Health Sciences Center. Lubbock, TX, USA
Thomas Leung. Division of Neurology, Department of Medicine and Therapeutics, The Chinese University of Hong Kong. Hong Kong, Hong Kong
Lawrence Wong. Division of Neurology, Department of Medicine and Therapeutics, The Chinese University of Hong Kong. Hong Kong, Hong Kong
Vincent Mok. Department of Medicine and Therapeutics, The Chinese University of Hong Kong. Hong Kong, Hong Kong

Richard Choy. Department of Obstetrics and Gynecology, The Chinese University of Hong Kong. Hong Kong, Hong Kong
Christina Jern. Department of Pathology and Genetics, The Sahlgrenska Academy at University of Gothenburg, Institute of Biomedicine. Gothenburg, Sweden
Elena Lebedeva. Department of Neurology and Neurosurgery, The Urals State Medical University, International Headache Center 'Europe–Asia'. Yekaterinburg, Russia
Martin Farrall. Department of Cardiovascular Medicine, The Wellcome Trust Centre for Human Genetics. Oxford, UK
Xu Jiayuan. Department of Radiology, Tianjin Medical University (TIJMU). Tianjin, China
Keat Wei Loo. Tunku Abdul Rahman University, UTAR. Kampar Perak, Malaysia
Isabel Hostettler. Stroke Research Center, UCL Institue of Neurology. London, UK
Gabriel Rinkel. Department of Neurology and Neurosurgery, UMC Utrecht, Brain Center Rudolf Magnus. Utrecht, Netherlands
Anderson Goncalves. Universidade da Região de Joinville. Joinville, Brazil
Paulo Franca. Department of Medicine, Universidade da Região de Joinville–Univille. Joinville, Brazil
Norberto Cabral. Department of Medicine, Universidade da Região de Joinville (Univille). Joinville, Brazil
Iscia Cendes. Molecular Genetics Laboratory, Universidade Estadual de Campinas, Faculdade de Ciências Médicas. Campinas, Brazil
Caty Carrera. Neurovascular Research Laboratory, Vall d'Hebron Institut of Research, Neurology and Medicine Departments, Universitat Autònoma de Barcelona, Vall d'Hebrón Hospital, Stroke Pharmacogenomics and Genetics, Fundacio Docència i Recerca Mutua Terrassa. Barcelona, Spain
Israel Fernandez-Cadenas. Neurovascular Research Laboratory, Vall d'Hebron Institut of Research, Neurology and Medicine Departments, Universitat Autònoma de Barcelona, Vall d'Hebrón Hospital, Stroke Pharmacogenomics and Genetics, Fundacio Docència i Recerca Mutua Terrassa. Barcelona, Spain
Joan Montaner. Neurovascular Research Laboratory, Universitat Autònoma de Barcelona, Vall d'Hebron Institute of Research, Neurology Department, Hospital Vall d'Hebron. Barcelona, Spain
Helen Kim. Institute for Human Genetics. The Center for Cerebrovascular Research (CCR). University of California. San Francisco, CA, USA
Arndt Rolfs. Albrecht Kossel Institute, University Clinic of Rostock. Rostock, Germany
Mayowa Owolabi. Department of Neurology, University College Hospital. Ibadan, Nigeria
Reecha Sofat. Metabolism & Experi Therapeutics, Division of Medicine, Faculty of Medical Sciences, University College London. London, UK
Mark Bakker. University Medical Center Utrecht. Utrecht, Netherlands
Dominique PV de Kleijn. Laboratory of Experimental Cardiology, University Medical Center Utrecht, ICIN–Netherlands Heart Institute. Utrecht, Netherlands
Ynte Ruigrok. Department of Neurology and Neurosurgery, University Medical Center Utrecht, Brain Center Rudolf Magnus, The Dutch Parelsnoer Institute–Cerebrovascular accident (CVA) Study Group. Utrecht, Netherlands
Allard Hauer. Department of Neurology and Neurosurgery, University Medical Center Utrecht, Brain Center Rudolf Magnus. Utrecht, Netherlands
Sara L. Pulit. Department of Neurology, University Medical Center Utrecht, Brain Center Rudolf Magnus. Utrecht, Netherlands
Ale Algra. Department of Neurology and Neurosurgery, University Medical Center Utrecht, Brain Center Rudolf Magnus, Julius Center for Health Sciences and Primary Care, University Medical Center Utrecht. Utrecht, Netherlands
Sander W. van der Laan. Laboratory of Experimental Cardiology, University Medical Center Utrecht, Division of Heart and Lungs. Utrecht, Netherlands
Mary Macleod. Department of Medicine & Therapeutics, University of Aberdeen, King's College. Aberdeen, UK
George Howard. School of Public Health, University of Alabama at Birmingham. Birmingham, AL, USA

Hemant Tiwari. Department of Biostatistics, University of Alabama at Birmingham. Birmingham, AL, USA
Ryan Irvin. Department of Epidemiology, University of Alabama at Birmingham. Birmingham, AL, USA
Karen C. Albright. Neurology, University of Alabama Hospital, Spectrum Health – Butterworth Campus. Birmingham, AL, USA
Rodney Perry. Department of Epidemiology, University of Alabama School of Medicine. Birmingham, AL, USA
Chelsea Kidwell. Department of Neurology, University of Arizona. Tucson, AZ, USA
Aleksandra Pavlovic. Neurology Clinic, Clinical Center of Serbia, University of Belgrade, Faculty of Medicine. Belgrade, Serbia
Christophe Tzourio. University of Bordeaux, INSERM U1219. Bordeaux, France
Murali Sargurupremraj. INSERM U1219 Bordeaux Population Health Research Center, University of Bordeaux. Bordeaux, France
Sabrina Schilling. University of Bordeaux. Bordeaux, France
Alessandro Padovani. Department of Clinical and Experimental Sciences, University of Brescia. Brescia, Italy
Alessandro Pezzini. Department of Clinical and Experimental Sciences, University of Brescia, Neurology Clinic. Brescia, Italy
Foad Abd-Allah. Department of Neurology, University of Cairo. Cairo, Egypt
Charles DeCarli. Alzheimer's Disease Center, University of California Davis. Davis, CA, USA
Yuqi Zhao. Department of Integrative Biology and Physiology, University of California. Los Angeles, CA, USA
David Liebeskind. Department of Neurology, University of California. Los Angeles, CA, USA
Matthew Traylor. Stroke Research Group, Division of Clinical Neurosciences, University of Cambridge. Cambridge, UK
Rhea Tan. Department of Clinical Neurosciences, University of Cambridge. Cambridge, UK
John Danesh. MRC/BHF Cardiovascular Epidemiology Unit, Department of Public Health and Primary Care, University of Cambridge, The National Institute for Health Research Blood and Transplant Research Unit in Donor Health and Genomics, Wellcome Trust Sanger Institute. Cambridge, UK
Susanna C. Larsson. Department of Clinical Neurosciences, Neurology Unit, University of Cambridge. Cambridge, UK
Loes Rutten-Jacobs. Stroke Research Group, University of Cambridge, Division of Clinical Neurosciences, DZNE. Cambdrige, UK
Amanda Donatti. School of Medical Sciences, FCM, University of Campinas, UNICAMP. São Paulo, Brazil
Wagner Avelar. Department of Neurology, University of Campinas, UNICAMP. São Paulo, Brazil
Joseph Broderick. Gardner Neuroscience Institute, Comprehensive Stroke Center, University of Cincinnati. Cincinnati, OH, USA
Daniel Woo. University of Cincinnati College of Medicine. Cincinnati, OH, USA
Brett Kissela. Department of Neurology and Rehabilitation Medicine, University of Cincinnati Gardner Neuroscience Institute, Comprehensive Stroke Center. Cincinnati, OH, USA
Laura Garcia Ibenez. Institute for Cancer Genetics, University of Columbia. New York, NY, USA
Rustam Salman. Centre for Clinical Brain Sciences, University of Edinburgh. Edinburgh, UK
Cathie Sudlow. Centre for Medical Informatics, Usher Institute, University of Edinburgh. Edinburgh, UK
Kristiina Rannikmäe. Centre for Medical Informatics, Usher Institute, University of Edinburgh. Edinburgh, UK
Caitrin Wheeler McDonough. College of Pharmacy, University of Florida. Gainesville, FL, USA
Scott Silliman. Department of Neurology, University of Florida College of Medicine. Jacksonville, FL, USA
Oyunbileg Magvanjav. Department of Pharmacotherapy and Translational Research and Center for Pharmacogenomics, University of Florida, College of Pharmacy. Gainesville, FL, USA
Tom van Agtmael. Insititute of Cardiovascular and Medical Sciences, University of Glasgow. Glasgow, UK
Matthew Walters. School of Medicine, Dentistry and Nursing, University of Glasgow. Glasgow, UK
Martin Söderholm. Bioinformatics Core Facility, University of Gothenburg. Gothenburg, Sweden

Erik Lorentzen. Bioinformatics Core Facility, University of Gothenburg. Gothenburg, Sweden
Sandra Olsson. University of Gothenburg. Gothenburg, Sweden
Tara Stanne. University of Gothenburg. Gothenburg, Sweden
Martina Olsson. University of Gothenburg. Gothenburg, Sweden
Kazuma Nakagawa. University of Hawaii, John A. Burns School of Medicine, Department of Neurology, The Queen's Medical Center. Honolulu, HI, USA
Rufus Akinyemi. Institute for Advanced Medical Research and Training, University of Ibadan, College of Medicine. Ibadan, Nigeria
Ioana Cotlatciuc. Institute of Cardiovascular Research Royal Holloway, University of London. London, UK
Jeff O'Connell. Medicine department, University of Maryland, Animal Genetics Improvement Laboratory, United States Department of Agriculture. Baltimore, MD, USA
Mary Sparks. Department of Neurology, University of Maryland. Baltimore, MD, USA
John Sorkin. Department of Neural and Pain Sciences, University of Maryland. Baltimore, MD, USA
Patrick McArdle. Division of Endocrinology, Diabetes and Nutrition, University of Maryland School of Medicine. Baltimore, MD, USA
Tushar Dave. Bioinformatics, University of Maryland School of Medicine. Baltimore, MD, USA
Colin Stine. Epidemiology & Public Health, University of Maryland School of Medicine. Baltimore, MD, USA
Steven Kittner. Department of Neurology, University of Maryland School of Medicine and Baltimore VAMC. Baltimore, MD, USA
Jill Naylor. University of Melbourne. Melbourne, Australia
Devin Brown. Department of Neurovascular, University of Michigan Medical School. Ann Arbor, MI, USA
Mario Di Napoli. Department of Neurology, University of Naples. Naples, Italy
Rose Du. Comprehensive Cancer Center, University of New Mexico. Albuquerque, NM, USA
Tobias B. Kulik. Neurology, University of New Mexico. Albuquerque, NM, USA
John Attia. School of Medicine and Public Health, University of Newcastle. New Lambton, Australia
Shahbaz Zamani. University of Newcastle. Newcastle, Australia
James E Faber. Cell Biology and Physiology, University of North Carolina. Chapel Hill, NC, USA
Peter Rothwell. Nuffield Department of Clinical Neurosciences, University of Oxford. Oxford, UK
Iona Millwood. Nuttfield Department of Population Health, University of Oxford. Oxford, UK
Elsa Valdés Márquez. CTSU–Clinical Trial Service Unit and Epidemiological Studies Unit, University of Oxford. Oxford, UK
Michelangelo Mancuso. Department of Neurology, University of Pisa. Pisa, Italy
Doralina Brum Souza. Department of Neurology, University of Sao Paulo. Ribeirao Preto, Brazil
Ranil de Silva. Genetic, Diagnostic and Research Laboratory, Department of Anatomy, University of Sri Jayewardenepura, Faculty of Medical Sciences. Nugegoda, Sri Lanka
Riina Vibo. Department of Neurology and Neurosurgery, University of Tartu. Tartu, Estonia
Janika Korv. Department of Neurology and Neurosurgery, University of Tartu. Tartu, Estonia
Jane Maguire. Faculty of Health, University of Technology Sydney. Ultimo, Australia
Myriam Fornage. Brown Foundation Institute of Molecular Medicine // Human Genetics Center, University of Texas Health Science Center at Houston. Houston, TX, USA
Kachikwu Illoh. University of Texas Medical School. Houston, TX, USA
Dianna Milewicz. Division of Medical Genetics, University of Texas Health Science Center, McGovern Medical School, Department of Internal Medicine, University of Texas Health Science Center, McGovern Medical School. Houston, TX, USA
Jennifer Majersik. Neurology, University of Utah. Salt Lake City, UT, USA
Adam DeHavenon. Department of Neurology, University of Utah. Salt Lake City, UT, USA
Yashar Kalani. Neurobiology and Anatomy, University of Utah School of Medicine. Salt Lake City, UT, USA
Matthew Alexander. Radiology and Imaging Sciences, University of Utah School of Medicine, Neurosurgery, University of Utah School of Medicine. Salt Lake City, UT, USA

Mary Cushman. Department of Hematology and Oncology, University of Vermont, Medical Center. Colchester, VT, USA
Michele Sale. Center for Public Health Genomics, University of Virginia. Charlottesville, VA, USA
Andrew Southerland. Departments of Neurology and Public Health Sciences, University of Virginia. Charlottesville, VA, USA
Debra Owens. Newborn Intensive Care Units, University of Virginia Children's Hospital. Charlottesville, VA, USA
Keith Keene. Center for Public Health Genomics, University of Virginia School of Medicine. Charlottesville, VA, USA
Stephen Rich. Center for Public Health Genomics, University of Virginia School of Medicine. Charlottesville, VA, USA
Bruce Psaty. Cardiovascular Health Research Unit, Department of Medicine, University of Washington, Department of Epidemiology, University of Washington. Seattle, WA, USA
Will Longstreth. Department of Epidemiology, University of Washington, Department of Neurology, University of Washington. Seattle, WA, USA
Masharip Atadzhanov. Department of Neurology, University Teaching Hospital. Lusaka, Zambia
Stacey Quintero Wolfe. Wake Forest Baptist Health. Winston-Salem, NC, USA
Carl Langefeld. Center for Public Health Genomics and Department of Biostatistical Sciences, Wake Forest School of Medicine. Winston-Salem, NC, USA
Cheryl Bushnell. Department of Neurology, Wake Forest University Medical Center. Winston-Salem, NC, USA
Carlos Cruchaga. Department of Psychiatry, The Hope Center Program on Protein Aggregation and Neurodegeneration (HPAN), Washington University School of Medicine, Department of Developmental Biology, Washington University School of Medicine. St. Louis, MO, USA
Jan Konrad. Department of Psychiatry, The Hope Center Program on Protein Aggregation and Neurodegeneration (HPAN), Washington University, School of Medicine. St. Louis, MO, USA
Junfeng Liu. West China Medical School, Sichuan University. Sichuan, China
Kevin Sheth. Department of Neurology, Neuro Intensive Care & Neurological Emergencies, Yale School of Medicine. New Haven, CT, USA
Guido Falcone. Department of Neurology, Yale University School of Medicine, Program in Medical and Population Genetics, The Broad Institute of Harvard and MIT. New Haven, CT, USA
Kathleen Donahue. J. Philip Kistler Stroke Research Center, Massachusetts General Hospital. Boston, MA, USA
